# UV induced reversible chain extension of 1-(2-anthryl)-1-phenylethylene functionalized polyisobutylene

**DOI:** 10.1080/15685551.2017.1382028

**Published:** 2017-10-16

**Authors:** Cimen Ozguc Onal, Turgut Nugay

**Affiliations:** ^a^ Chemistry Department, Polymer Research Center, Bogazici University, Istanbul, Turkey

**Keywords:** Anthracene, photochemistry, reversible chain-extension, polyisobutylene, cationic polymerization

## Abstract

The synthesis of novel 1-(2-anthryl)-1-phenylethylene (APE) di-telechelic polyisobutylenes is described. Utilization of a difunctional cationic initiator and the *in situ* addition of the non-homopolymerizable APE lead to the formation of di-anthryl telechelic polyisobutylenes. Products were characterized by ^1^H NMR spectroscopy and Size Exclusion Chromatography. The polymers were UV irradiated at 365 and 254 nm and the reversible photocycloaddition of anthryl moieties was investigated. The chain extension of di-anthryl telechelic PIBs through photocoupling at 365 nm produced higher molecular weight products from low molecular weight precursors. The effect of precursor polymer concentration on the degree of chain extension was investigated, and intermolecular interactions leading to the formation of tetramers was observed. The photocoupled products were UV irradiated at 254 nm to induce the reversal of photocycloaddition of anthryl groups and to follow the consequent photoscission of polymers.

## Introduction

The reversible dimerization behavior of anthracene and its derivatives has been studied extensively since the nineteenth century. Today it is well known that anthracene molecules have the ability to photodimerize by UV irradiation above 300 nm via a [4π + 4π] cycloaddition reaction through the 9- and 10-positions; the resulting photodimers can be reverted back to original state either thermally at an elevated temperature or via UV irradiation with a wavelength below 300 nm [1,2]. Advantageous aspects of this phenomenon have been widely investigated via utilization of various anthracene derivatives [2–4] and anthryl functionalized polymers [5–26]. The photocycloaddition property has been applied to form crosslinked polymers systems [5–9] and used in different application areas such as controlled release studies [10,11], pattering of polymer films [12–15], self-healing materials [16,17], and hydrogels [18,19]. Anthracene end-functionalized polymers were subjected to photodimerization in order to perform chain extension [20], to obtain block copolymers [21], and to form cyclic products [22–26].

In this study, inclusion of the photosensitive anthracene molecule was achieved through the utilization of 1-(2-anthryl)-1-phenylethylene (APE). APE was first synthesized and incorporated into copolymer structure as a fluorescent labelling agent, drawing advantage from the fact that APE does not homopolymerize [27,28]. This property enabled the molecule to be used as an end-capping agent, and in further studies the insertion of APE molecules to polymer chain ends allowed the synthesis of various anthryl functionalized block copolymers [29–34].

The present work demonstrates the synthesis of a novel APE functionalized isobutylene polymer via cationic polymerization. Chain end modification of polyisobutylene (PIB) was performed by *in situ* addition of APE; benefiting from the non-homopolymerizable nature of the molecule to obtain end functionalization by single APE moieties. Utilization of a difunctional cationic initiator in the process enables the synthesis of di-anthryl telechelic PIB chains. Employment of other 1,1-diarylethylene compounds for the end functionalization of PIB was achieved in previous studies [35–42]. Quantitative end-capping of PIB chains was performed by using 1,1-diphenylethylene (DPE) [35–39] and derivatives of DPE; [40–42] in consideration of the fact that the reaction of PIB chain end with DPE results in the formation of a more stable carbocation and the inability of the molecule to homopolymerize which allows the addition of only one DPE unit [37]. This strategy has proved to be advantageous for the synthesis of several block copolymers [38–42]. The current work is aimed to examine the chain extension of di-anthryl telechelic PIBs through photocycloaddition coupling reactions to reach higher molecular weight products from low molecular weight precursors.

## Experimental

### Materials

Anthracene, acetic anhydride, aluminum chloride (AlCl_3_), diethyl ether (DEE), methanol (CH_3_OH), phosphorous pentoxide, acetic acid (CH_3_COOH), sulfuric acid (H_2_SO_4_), magnesium sulfate (MgSO_4_), hydrochloric acid (HCl), sodium bicarbonate (NaHCO_3_), titanium tetrachloride (TiCl_4_), calcium hydride (CaH_2_), benzophenone were purchased from Merck at the highest purity available and used as received. Sodium (Na) metal was purchased from Alfa Aesar and used as received. Isobutylene (IB) was purchased from Scott Specialty Gases and was dried by passing through columns packed with anhydrous calcium sulfate, cobalt chloride, moisture sensitive color indicator, silica gel and condensed into a N_2_ atmosphere reactor at −40 °C prior to use.

Phenylmagnesium bromide (PhMgBr, 3M in DEE solution) was purchased from Sigma Aldrich and used as received.

Dichloromethane (DCM), *n*-hexane, tetrahydrofuran (THF) and 2,6-di-tert-butylpyridine (DtBP) were purchased from Merck.

DCM and *n*-hexane were initially refluxed over calcium hydride; followed by distillation in reduced pressure over sodium mirror and phosphorous pentoxide, respectively. THF was distilled over sodium metal and benzophenone. DtBP was purified by distillation over calcium hydride.

### Synthesis of 1-(2-anthryl)-1-phenylethylene (APE)

APE was synthesized as previously described [27]. Initially, the Friedel-Crafts reaction was utilized for the synthesis of 2-acetylanthracene intermediate via addition of acetic anhydride to anthracene in the presence of AlCl_3_ in nitrobenzene. The product was recrystallized from benzene/hexane mixture and purified by further extractions. In the next step, of 2-acetylanthracene (11.95 g, 54.3 mmol) was dissolved in dry toluene (350 ml) and 35 ml of 3 M phenylmagnesium bromide in diethyl ether was added dropwise. The reaction mixture was refluxed for 12 h and then poured into crushed ice (460 ml) and concentrated hydrochloric acid (46 ml) mixture. Extraction was performed with toluene, product was dried over magnesium sulfate and the solvent was evaporated. The crude product was dissolved in 240 ml of hot acetic acid and 0.2 ml concentrated sulfuric acid was added. The mixture was cooled; the crystalline product was collected, washed with cold acetic acid and dried. The monomer was further purified by recrystallization.

^1^H NMR (CDCl_3_): δ (ppm) = 8.41(s, 1H), 8.37 (s, 1H), 8.05–7.90 (m, 4H), 7.53–7.33 (m, 8H), 5.67 (s, 1H), 5.57 (s, 1H).

### Synthesis of 5-tert-butyl-1,3-bis(2-chloro-2-propyl)benzene (t-Bu-m-DiCumCl)

The difunctional cationic initiator t-Bu-m-DiCumCl was synthesized in a similar manner as previously described [43,44].

### Synthesis of di-anthryl telechelic polyisobutylene

**Figure 1.**
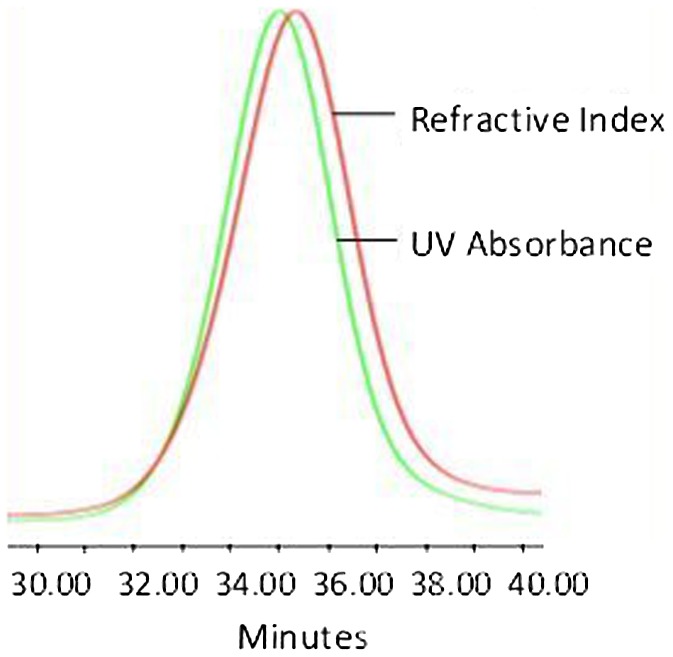
APE-PIB-APE-1 SEC data recorded by UV absorbance and refractive index detectors.

**Figure 2.**
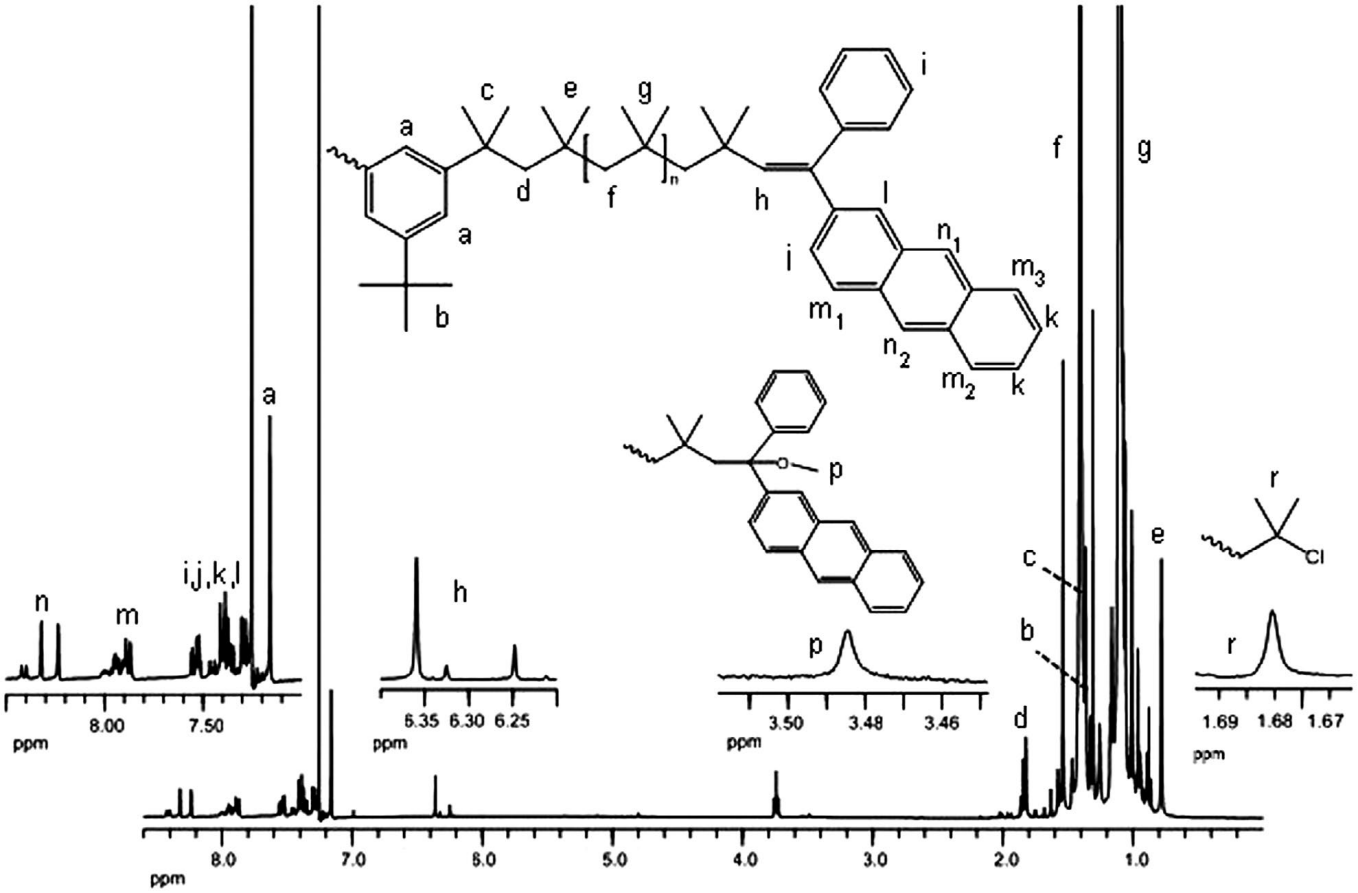
^1^H NMR spectrum of APE-PIB-APE-1.

A representative polymerization procedure of APE-PIB-APE carried out by cationic polymerization is as follows. A 500 ml one neck round bottom flask equipped with a magnetic stirrer bar and a rubber septum was connected to the vacuum line and flamed under vacuum and charged with nitrogen gas. The polymerization was performed in freshly distilled *n*-hexane/DCM (60:40%) solvent mixture at −80 °C under nitrogen atmosphere; with t-Bu-m-DiCumCl/TiCl_4_ initiating system and DtBP as the proton trap. Hexane (51 ml), DCM (33 ml) and t-Bu-m-DiCumCl (0.4765 g, 1.66 × 10^−3^ mol) were transferred into the reactor. 0.2 ml (8.3 × 10^−5^ mol) of a 5 ml stock solution of DtBP in hexane was added via syringe equipped with steel capillary and then the reactor was cooled to −80 °C. Isobutylene (8 ml, 1.08 × 10^−1^ mol) was condensed into a previously flamed and nitrogen filled graduated cylinder at −40 °C and added to the reactor via capillary. After the transfer of TiCl_4_ (3.66 ml, 3.33 × 10^−2^ mol) the polymerization was conducted −80 °C for 1 h. Then, a solution of APE (1.12 g, 4 × 10^−3^ mol in 60 ml DCM) added into the reactor via capillary and the reaction was continued for 3 h. Termination was performed by the addition of 5 ml methanol and the polymer solution was precipitated into excess methanol. The obtained polymer was dissolved in hexane, and washed with 5% aqueous sodium bicarbonate and water. The organic phase was dried overnight over magnesium sulfate, filtered through fine sintered glass and the solvent was evaporated on rotary evaporator. Then, the polymer was redissolved in a small amount of hexane and precipitated into acetone twice in order to remove excess APE. Finally, the polymer was further dried on high vacuum line. Figures 1 and S1 show the Size Exclusion Chromatography (SEC) traces of the synthesized polymers APE-PIB-APE-1 and APE-PIB-APE-2; the corresponding ^1^H NMR spectra are given in Figures 2 and S2, respectively.

**Figure 3.**
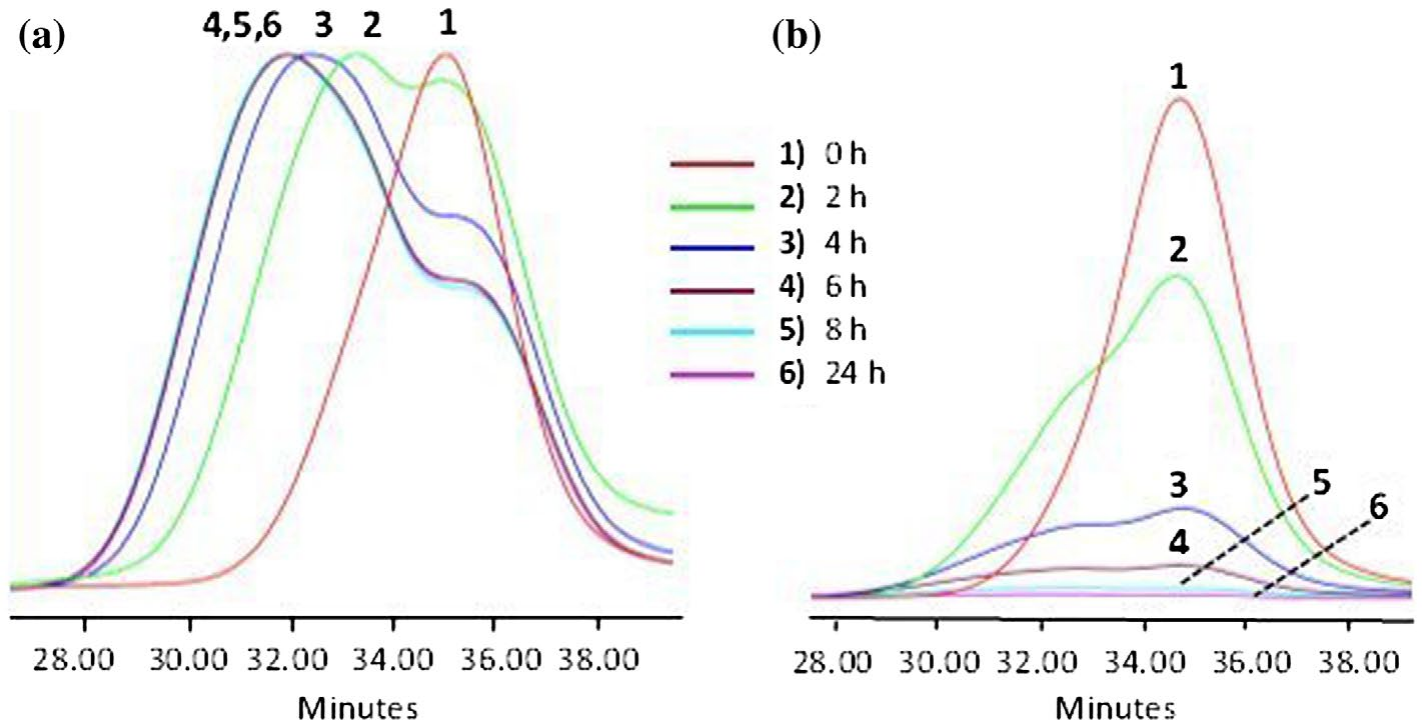
SEC data of APE-PIB-APE-1 recorded by (a) RI and (b) UV absorbance detectors: before irradiation (1), after irradiation at 365 nm for 2 h (2), after irradiation at 365 nm for 4 h (3), after irradiation at 365 nm for 6 h (4), after irradiation at 365 nm for 8 h (5), after irradiation at 365 nm for 24 h (6).

**Figure 4.**
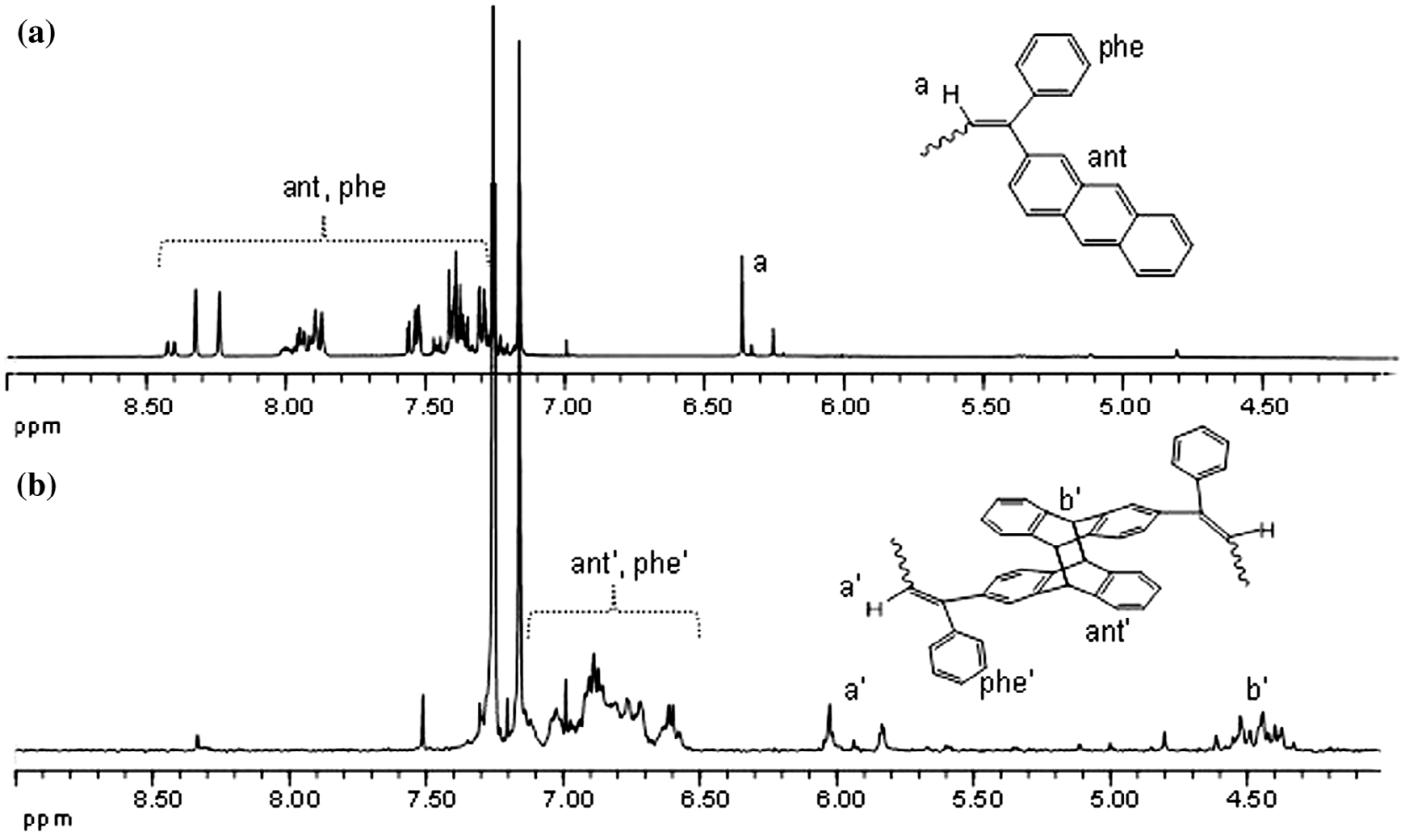
^1^H NMR spectrum of APE-PIB-APE-1 (a) before irradiation (b) after irradiation at 365 nm.

**Figure 5.**
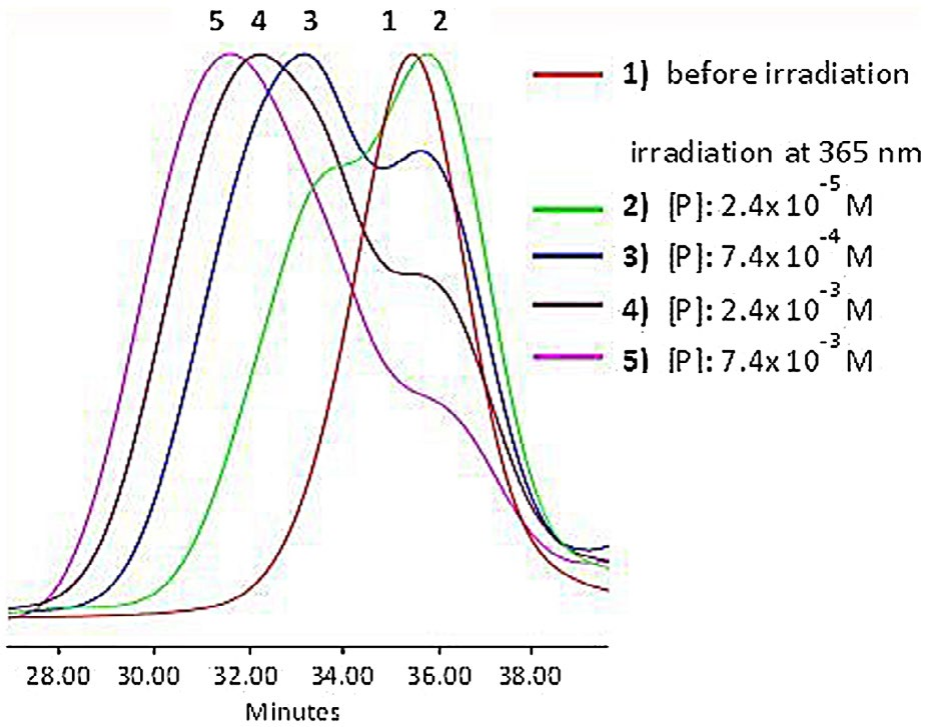
SEC data of APE-PIB-APE-1 recorded by RI detector; before irradiation (1) and after irradiation at 365 nm for 24 h with different precursor polymer concentrations: [P]: 2.4 × 10^−5^ M (2), 7.4 × 10^−4^ M (3), 2.4 × 10^−3^ M (4), 7.4 × 10^−3^ M (5).

**Figure 6.**
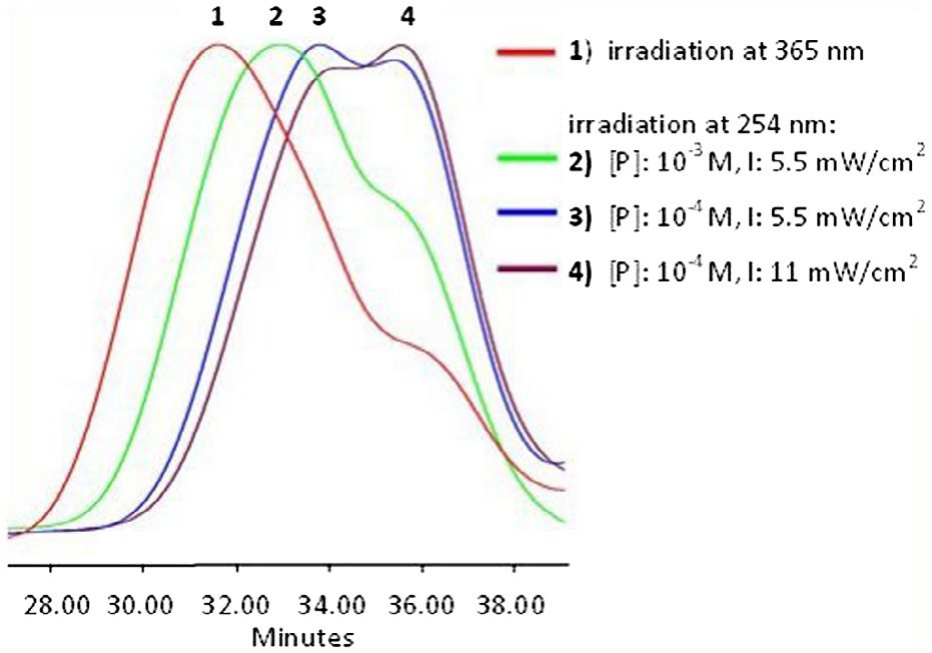
SEC data of APE-PIB-APE-1 recorded by RI detector; after irradiation at 365 nm for 24 h (1), followed by irradiation at 254 nm for 2 h with different precursor polymer concentrations and light intensities: [P]: 10^−3^ M, I: 5.5 mW/cm^2^ (2), [P]: 10^−4^ M, I: 5.5 mW/cm^2^ (3), [P]: 10^−4^ M, I: 11 mW/cm^2^ (4).

**Figure 7.**
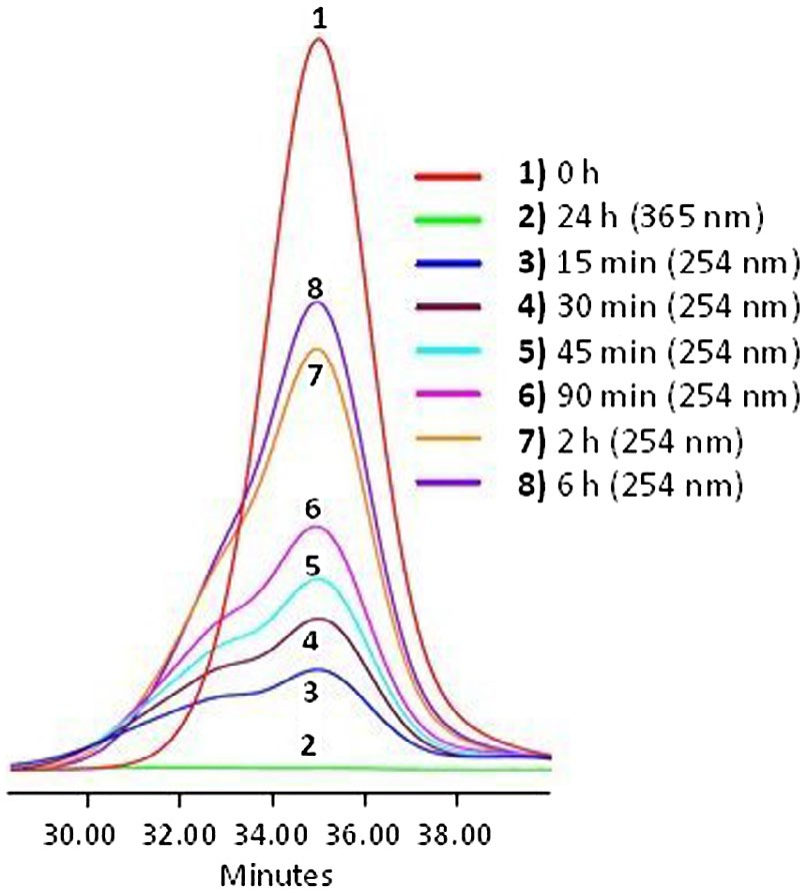
Absorbance of APE-PIB-APE-1 at 365 nm recorded by SEC UV detector; before irradiation (1), after irradiation at 365 nm for 24 h (2), followed by irradiation at 254 nm (3–8).

**Figure 8.**
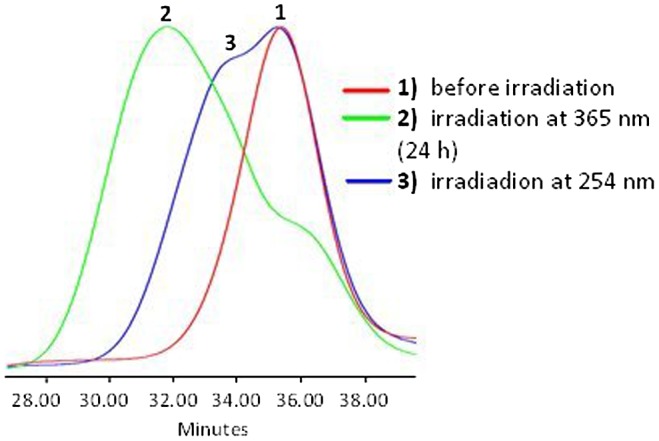
SEC data of APE-PIB-APE-1 recorded by RI detector; before irradiation (1), after irradiation at 365 nm for 24 h (2), followed by irradiation at 254 nm for 6 h (3).

**Figure 9.**
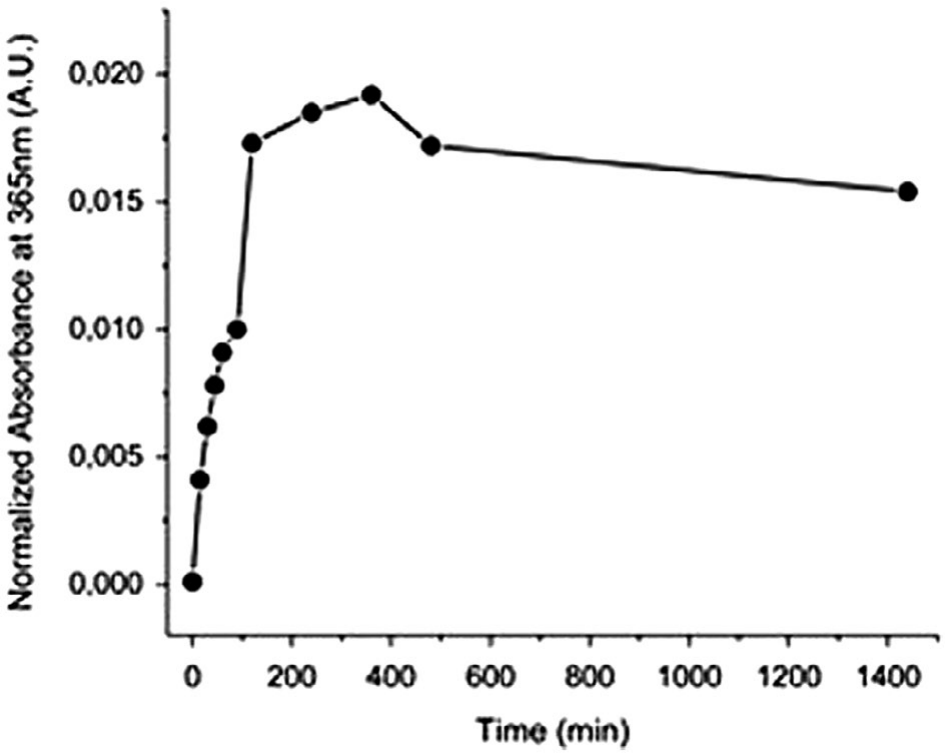
Absorbance at 365 nm vs. time under irradiation at 254 nm, recorded by SEC UV detector (the sample APE-PIB-APE-1 had been previously exposed to 365 nm UV light for 24 h for maximum photocycloaddition).

**Figure 10.**
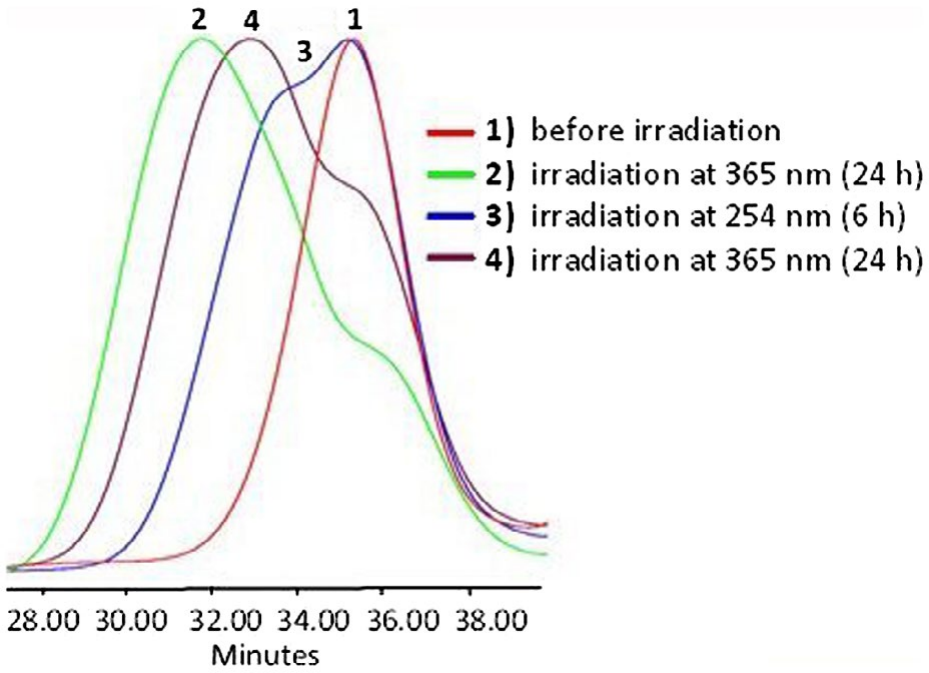
SEC data of APE-PIB-APE-1 recorded by RI detector; before irradiation (1), after irradiation at 365 nm for 24 h (2), followed by irradiation at 254 nm for 6 h (3), followed by irradiation at 365 nm for 24 h (4).

### UV irradiation experiments

A custom made UV chamber was utilized for the UV irradiation experiments of the synthesized polymers. Photocycloaddition reactions of the anthryl functionalized polymers were conducted with low-pressure mercury-vapor fluorescent lamps (8 W), with maximum emission wavelength of 365 nm. The intensity of light was measured as 10 mW/cm^2^, by using UVA/B Light Meter. Photoscission reactions were performed with low-pressure mercury-vapor fluorescent lamps (8 W), with maximum emission wavelength of 254 nm. The intensity of light was measured as 5.5 or 11 mW/cm^2^, by using UVC Light Meter. The reactions were carried out in crimp sealed vials under nitrogen atmosphere. Initially, the polymers to be used were weighed into vials which were then sealed, evacuated under vacuum and purged with nitrogen gas. Then a required amount of distilled THF was transferred into the sealed vial through capillary under N_2_ atmosphere. The reactor vial was placed into the UV chamber and the polymer solution was stirred throughout the reaction. Sampling at different time intervals was done under nitrogen atmosphere.

### Characterization

^1^H NMR spectroscopy was performed on Varian Gemini 400 MHz spectrometer at room temperature with deuterated chloroform (δ CDCl_3_: 7.26 ppm) as solvent.

The number average molecular weights (M_n_) and polydispersities (PDI) of the polymers were determined by Size Exclusion Chromatography (SEC) at 30 °C using Waters Isocratic HPLC Pump with Waters 2414 Refractive Index (RI) Detector, Waters 2487 UV absorbance detector and four Waters Styragel columns (HR 3, HR 4, HR 4E and HR 5E). Distilled THF was used as the mobile phase at a flow rate of 0.35 mL THF/min. Polystyrene standards used for calibration were in the range of 400–180,000. Samples were injected using 100 μL Hamilton syringe. For the detection of anthracene moieties, UV absorbance detector was adjusted to 365 nm.

## Results and discussion

Di-anthryl telechelic PIBs were synthesized using the aforementioned method and the corresponding SEC data for the synthesized polymers APE-PIB-APE-1 and APE-PIB-APE-2 are demonstrated in Figures 1 and S1, respectively. UV absorbance detector at 365 nm was utilized alongside the refractive index detector in order to monitor the presence of the anthracene moieties on the polymer chains. The overlap in the chromatograms indicates that the polymer chains were successfully functionalized with APE.

The ^1^H NMR spectrum of APE-PIB-APE-1 (Figure 2) displays the characteristic polyisobutylene peaks, with methylene protons of the backbone appearing between 1.35 and 1.46 ppm and methyl protons between 1.03 and 1.22 ppm. The signals at 7.2–8.5 ppm correspond to the anthryl and phenyl groups of APE moieties, which indicate successful end functionalization with 97.2% yield. The remaining 2.8% is attributed to tert-chloride chain end, which is observed by the *gem*-dimethyl protons at 1.68 ppm. The peak at 3.5 ppm corresponds to the methoxy protons of 1-(2-anthryl)-1-methoxy-1-phenyl chain end with 3.8% yield. The signals at 6.2–6.4 ppm arise from the formation of vinylic hydrogen of 2-(2-anthryl)-2-phenlyvinyl type chain end, with 93.4% yield, which is expected due to the termination conditions; addition of methanol to the highly acidic reaction mixture induces the formation of elimination product [39]. The higher molecular weight polymer APE-PIB-APE-2 shows 88.1% end functionalization calculated from ^1^H NMR spectrum (Figure S2). For both polymers, the methyl protons of polymer backbone (1.03–1.22 ppm) and the methyl protons of the first isobutylene units attached to the bifunctional initiator (0.78 ppm) were utilized for the calculation of M_n_ values (Table 1).

**Table 1.** Characterization data of synthesized polymers.

Sample name	M_n_^a^ (g/mol)	M_n_^b^ (g/mol)	M_p_^b^	PDI^b^	APE %^a^
APE-PIB-APE-1	6357	6893	7456	1.20	97.2
APE-PIB-APE-2	17,545	18,005	20,288	1.18	88.1

^a^ Calculated by ^1^H NMR.

^b^ Determined by SEC.

**Scheme 1.**
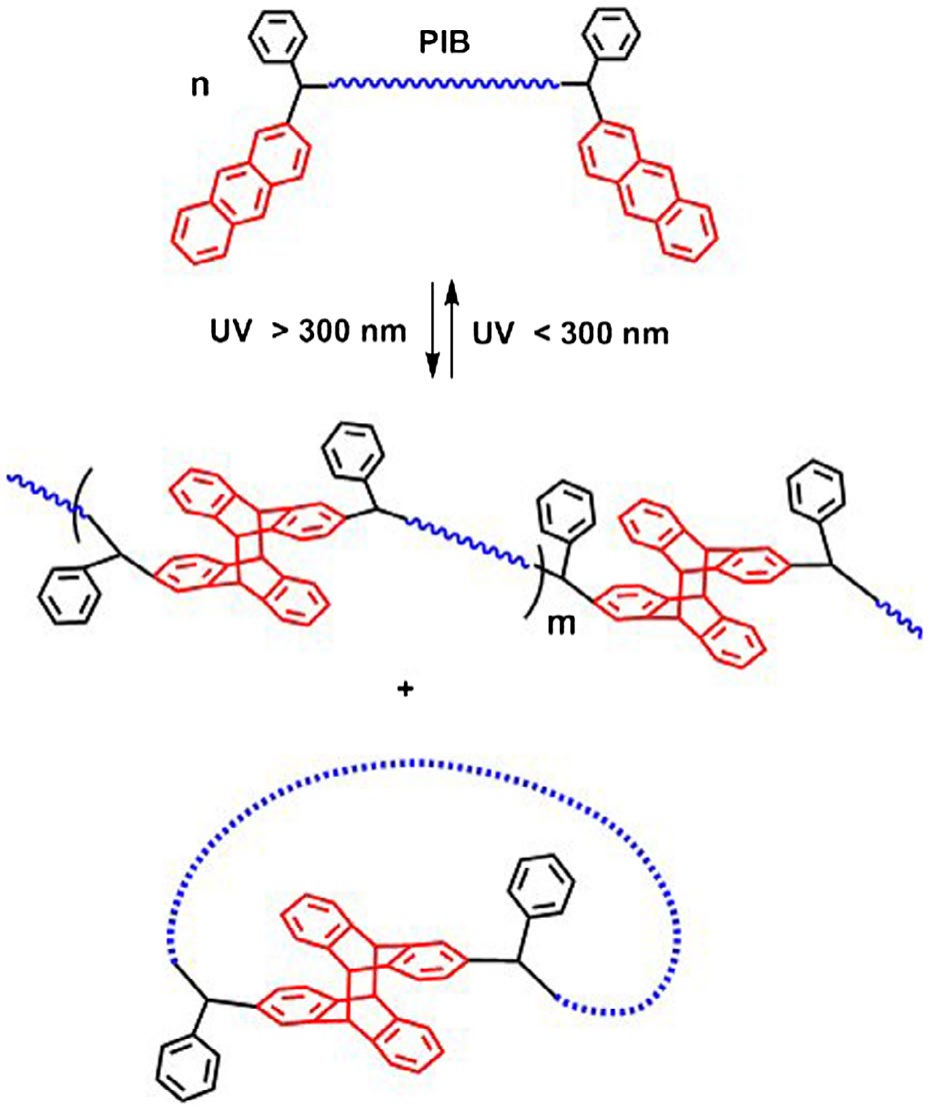
Photocycloaddition reactions of di-anthryl telechelic polymers.

The synthesized di-anthryl telechelic PIBs were then utilized in UV irradiation experiments in order to examine the photocycloaddition reactions that induce chain extension and formation of higher molecular weight products. Upon irradiation with UV light at 365 nm, polymer chains having APE units at the chain ends would couple via [4π + 4π] cycloaddition of anthracene moieties (Scheme 1). Depending on the experimental conditions such as concentration, molecular weight and solvent; the polymer chains may undergo intermolecular interactions leading to chain extension or intramolecular interactions that result in the formation of cyclic polymers. The chain extension process is predominant in the case of high polymer concentrations and utilization of high molecular weight polymers, since both conditions increase the probability of intermolecular interactions among polymer chains [45].

Chain extension of APE functionalized polymers can be monitored by SEC analysis through the increase in the molecular weight with respect to reaction time. Subsequent decrease in absorbance in the UV chromatograms at 365 nm by the photocoupling of anthracene units can also be observed; owing to the fact that photodimerization of the anthracene units cause a disruption in the conjugation of the π system and the resulting photodimers no longer absorb light of wavelengths greater than 300 nm [3]. The photocycloaddition reactions of anthracene moieties should be conducted under inert atmosphere in order to prevent the formation of endoperoxides that occurs in the presence of air [5,6].

Figure 3 shows the SEC curves of APE-PIB-APE-1 prior to and after UV irradiation at 365 nm for different reaction times. As the anthracene moieties at the chain ends undergo intermolecular photocycloaddition, the molecular weight of polymer chains increases which is evident in the shift to shorter retention time in SEC analysis (Line 1 vs. 6 in Figure 3). Concurrently, absorbance of the polymer samples monitored by the UV detector at 365 nm decreases gradually (Figure 3(b)) following the depletion of anthracene units by photocycloaddition reaction.

^1^H NMR spectrum of APE-PIB-APE-1 prior to and after irradiation at 365 nm is demonstrated in Figure 4. The comparison of the two spectra shows that the anthryl and phenyl proton peaks of the original polymer chain end at 7.2–8.5 ppm disappear upon UV treatment and new peaks corresponding to the aromatic groups of the cycloaddition product appear at 6.5–7.1 ppm. The bridgehead protons of the coupled structure are also visible at 4.3–4.6 ppm, which indicates successful photodimerization of anthracene units.

To gain insight on the effect of concentration on the degree of chain extension, four samples of APE-PIB-APE-1 with different precursor polymer concentrations ([P]: 2.4 × 10^−5^ M, 7.4 × 10^−4^ M, 2.4 × 10^−3^ M, 7.4 × 10^−3^ M) were subjected to UV irradiation of 365 nm. The formation of higher molecular weight peaks are observed in all four by the shift of the retention time to lower values, following the trend of increasing concentration as shown in Figure 5. The maximum M_n_ and M_p_ values reached after irradiation at 365 nm for 24 h are listed in Table S1, as well as the initial M_n_ and M_p_ value of APE-PIB-APE-1 for comparison.

It was observed that the sample of [P]: 7.4 × 10^−4^ M shows an approximately twofold increase in the M_p_ value compared to the original polymer within 24 h. Sample [P]: 2.4 × 10^−3^ M reaches a threefold M_p_ value; whereas in the most concentrated sample of [P]: 7.4 × 10^−3^ M, the average M_p_ value is approximately four times the original polymer. This indicates that precursor polymer concentration directly affects the degree of chain extension; higher amount of APE-PIB-APE-1 in solution allows the chains to undergo intermolecular interactions and leads to the formation of tetrameric structures in the most concentrated sample. On the other hand, the original polymer peak (Figure 5, Line 1) does not disappear within 24 h, meaning that not all polymer chains undergo extension. Furthermore, the M_p_ value of this peak shifts to longer retention time throughout the reaction. This shows that the remaining polymer units that are unable to participate in chain extension may undergo intramolecular interactions instead and form unicyclic products. These cyclic polymers appear at longer retention times compared to their linear counterparts, due to their compact structures and smaller hydrodynamic volumes [45–48]. The UV absorbance value of the abovementioned peak diminishes in time, which also points to the depletion of anthracene units via cyclization. The most distinct shift to longer retention time is observed for the relatively dilute sample (Figure 5, Line 2). This indicates that the formation of unicyclic product is prominent for the least concentrated sample as expected, since low polymer concentration increases the probability of intramolecular interactions [45,48]. The formation of a high molecular weight shoulder for this sample implies that chain extension could not be avoided even in the most dilute condition. However, it should be pointed out that cyclization occurs predominantly in dilute media, and chain extension does not go beyond the formation of a dimer.

APE-PIB-APE-2 was also subjected to UV irradiation at 365 nm, with precursor polymer concentration [P]: 6 × 10^−3^ M. Formation of higher molecular weight peak was observed, but the shift to higher molecular weights was not as prominent as the low molecular weight counterpart APE-PIB-APE-1. SEC RI data show only an approximately twofold increase in the M_p_ value with respect to the original polymer within 8 h (Figure S3), even at high precursor polymer concentration. Longer polymer chains tend to undergo physical restrictions and diffusion problems that reduce their mobility. It has been previously stated that the mobility of anthryl group is crucial to enable the photocycloaddition reaction [14,49], therefore a lesser degree of chain extension might be expected for the high molecular weight polymer sample.

Next, photoscission experiments were conducted by using the photocoupled samples of APE-PIB-APE-1, obtained by irradiation at 365 nm for 24 h, at concentrated conditions to yield the highest molecular weight starting products. Irradiation of these samples under UV light at 254 nm leads to reversal of the photocycloaddition reaction, converting the photocoupled APE units to original state and resulting in the photoscission of polymer chains (Scheme 1). Reactions were once again performed in THF under inert atmosphere. The process can be monitored via SEC analysis by the shift of polymer peak molecular weight to lower values and the increase in the UV absorbance at 365 nm arising from the recovery of anthracene moieties at the chain ends.

When two samples with different precursor polymer concentrations [P]: 10^−3^ M (Figure 6, Line 2) and [P]: 10^−4^ M (Figure 6, Line 3) were subjected to irradiation at 254 nm (I: 5.5 mW/cm^2^) for 2 h, the greater shift to lower molecular weight region took place with the relatively dilute sample (Figure 6, Line 3). Evidently, the original polymer was recovered via photoscission at a higher degree in dilute conditions, which provide enhanced mobility to the polymer chains and facilitate photocleavage [6,17]. When the intensity of light was increased to 11 mW/cm^2^ and the initial polymer concentration was adjusted to 10^−4^ M, SEC data showed the furthest shift of the molecular weight to lower values (Figure 6, Line 4).

The dynamics of the photoscission reaction was further investigated by examining the correlation between irradiation time and change in absorbance at 365 nm (Figure 7). SEC UV data demonstrates that the absorbance value increases with irradiation time due to the regeneration of anthryl moieties via photocleavage under 254 nm UV light. The highest recovery of APE units was observed upon 6 h irradiation at 254 nm (Figure 7, Line 8). When compared to the absorbance value of the nonirradiated initial polymer (Figure 7, Line 1) and the following decrease to minimum via irradiation at 365 nm (Figure 7, Line 2), the sample irradiated at 254 nm for 6 h exhibited a 78% recovery of anthryl units (Figure 7, Line 8) determined by comparison of the areas under the absorbance curves. SEC RI data is also consistent with these results, exhibiting a shift of polymer molecular weight to lower values due to chain scission (Figure 8, Line 3).

On the other hand as the sample was exposed to further UVC treatment, extending the irradiation time to 8 h and then 24 h, the absorbance value decreased (Figure 9). These results show that an optimum irradiation dose has been reached within 6 h beyond which the photocleavage and photocycloaddition reactions are at dynamic equilibrium. Similar phenomena have been reported in previous studies for both anthracene [15,18] and coumarin [50,51] functionalized polymeric systems; an equilibrium state between the dimeric and photocleaved species is observed upon 254 nm irradiation after the maximum absorbance recovery is reached. Prolonged irradiation at 254 nm also renders the anthryl moieties susceptible to irreversible photocoupling [15]. It has been reported that irradiation of anthryl groups to induce photodimerization and photoscission may lead to formation of side products and irreversibly crosslinked structures [5], mainly due to radical reactions, which occur most prominently in the presence of oxygen [52]. Therefore it is crucial to eliminate oxygen from the reaction medium as much as possible and work under inert conditions, as previously mentioned.

The photocleaved sample was then subjected to 365 nm irradiation once again in order to investigate the viability of the photocycloaddition of anthryl units. Upon 24 h exposure to 365 nm light a shift to shorter retention time was observed in SEC RI data (Figure 10, Line 4), indicating that the polymer chains are undergoing photocoupling again to form higher molecular weight products.

## Conclusions

Synthesis of di-anthryl telechelic polyisobutylene was accomplished successfully by cationic polymerization followed by *in situ* chain end functionalization with 1-(2-anthryl)-1-phenylethylene. Simultaneous SEC analysis by refractive index detector and UV absorbance detector at 365 nm was carried out for the detection of anthracene units on the polymer chains. ^1^H NMR spectroscopy was utilized to confirm the structure of products, exhibiting 97.2% end functionalization for APE-PIB-APE-1 and 88.1% for APE-PIB-APE-2.

UV irradiation experiments were performed on the synthesized di-anthryl telechelic PIBs with alternating wavelengths of 365 and 254 nm to investigate the reversible photocycloaddition property of anthracene moieties. Upon irradiation with 365 nm light, the polymer chains exhibited photocoupling via [4π + 4π] cycloaddition of anthryl units at chain ends leading to the formation of higher molecular weight products. The process was successfully followed by SEC method; through the shift of polymer molecular weights to shorter retention times on the RI detector and the subsequent decrease in the UV absorbance detector at 365 nm due to the depletion of anthryl units via photocycloaddition. It was observed that increasing the precursor polymer concentration enhanced the amount of chain extension for APE-PIB-APE-1. Higher molecular weight sample APE-PIB-APE-2 displayed an approximately twofold increase in the M_p_ value indicating the formation of dimers; whereas APE-PIB-APE-1 exhibited a greater degree of chain extension, attaining tetramer formation in relatively concentrated reaction conditions. The original polymer peak of APE-PIB-APE-1 did not disappear within 24 h irradiation and shifted to longer retention time throughout the reaction. These results show that in addition to some extent of chain extension, di-anthryl telechelic polymer chains participated in intramolecular interactions as well and formed unicyclic products, most prominently in dilute conditions.

The photocoupled products were then subjected to UV irradiation at 254 nm to investigate the reversibility of the photocycloaddition process, which converted the photocoupled anthryl moieties to original state and induced photoscission on the polymer chains. Upon irradiation with 254 nm light, shift of polymer peak molecular weight to lower values was observed by SEC RI detector arising from the formation of smaller precursor polymers. The recovery of anthryl units at the chain ends was followed through the increase in the SEC UV absorbance value at 365 nm. It was observed that dilute reaction conditions aided the reversion to original polymer structure via photoscission and an optimum irradiation dose was reached within 6 h to yield 78% recovery of anthryl units, determined by comparison of absorbance increase with respect to initial values. Re-irradiation of the photocleaved sample under 365 nm light showed the expected shift of polymer M_p_ to a higher value, displaying the reversibility of photocycloaddition process.

## Disclosure statement

No potential conflict of interest was reported by the authors.

## Funding

This work was supported by the Boğaziçi University Research Foundation [grant number 13B05D3].

## Supplemental data

Supplemental data for this article is available online at https://doi.org/10.1080/15685551.2017.1382028.

## Supplementary Material

TDMP_1382028_Supplementary_Material.pdf
