# Supplementary material for: UV induced reversible chain extension of 1-(2-anthryl)-1-phenylethylene functionalized polyisobutylene
Source: Des Monomers Polym. 2017 Oct 16;20(1):514–23. doi: 10.1080/15685551.2017.1382028 (PMC5784863; doi:10.1080/15685551.2017.1382028)
Supplement: TDMP_1382028_Supplementary_Material.pdf [file TDMP_A_1382028_SM7982.pdf]

Supporting Information

UV induced reversible chain extension of 1-(2-anthryl)-1-phenylethylene functionalized polyisobutylene

Cimen Ozguc Onal, Turgut Nugay

Chemistry Department, Polymer Research Center, Boğaziçi University, 34342, Bebek, Istanbul, Turkey

Correspondence to:

T. Nugay (Email: nugay@boun.edu.tr) (Phone: +902123596671)

C. Ozguc Onal (Email: cimenozguc@gmail.com) (Phone: +902123597135)

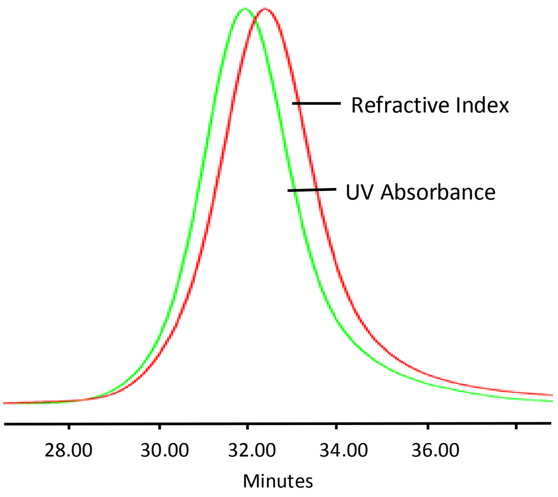

FIGURE S1. SEC data of APE-PIB-APE-2 recorded by UV absorbance and refractive index detectors.

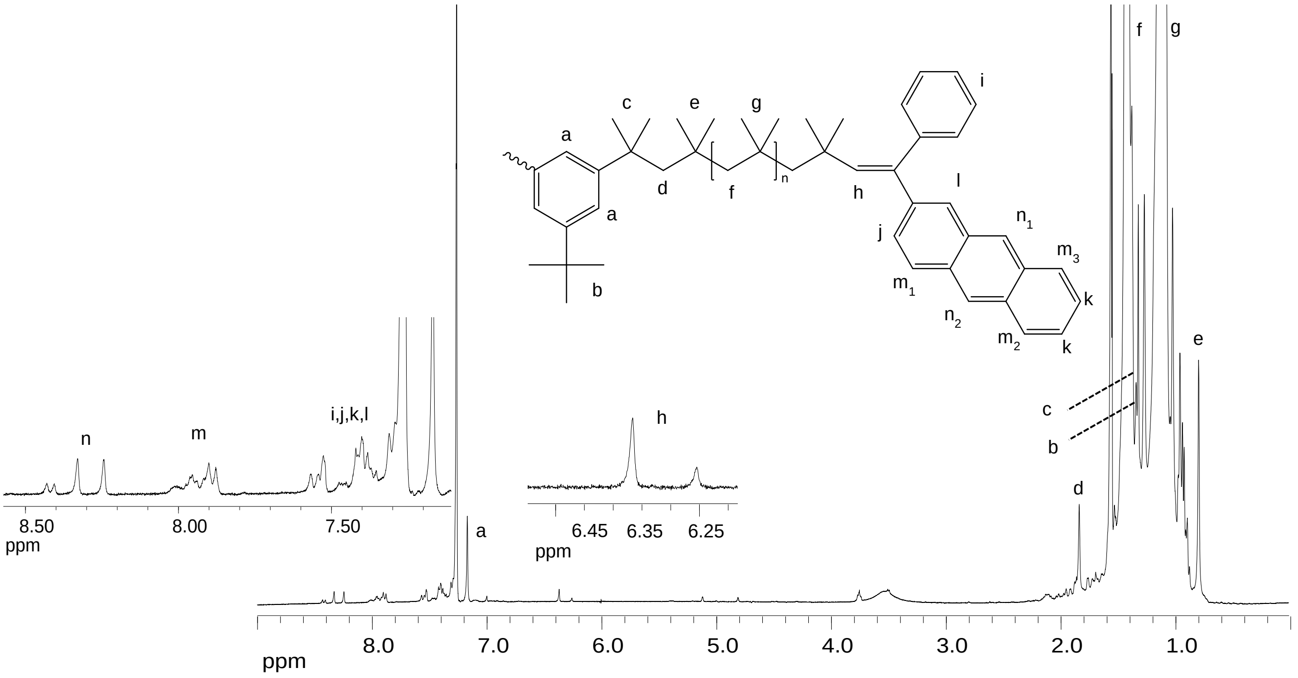

FIGURE S2. ^{1}H-NMR spectrum of APE-PIB-APE-2.

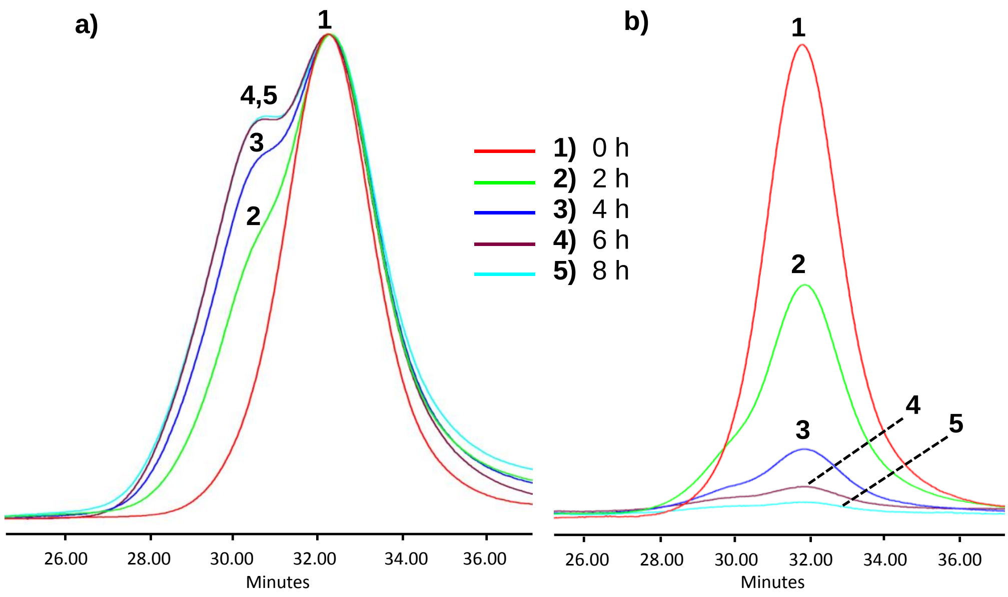

FIGURE S3. RI and UV SEC data of APE-PIB-APE-2 at specific reaction times.

Table S1. SEC RI data of APE-PIB-APE-1 irradiated at 365 nm for 24 h depicted in Figure 5.

	[P]: (M)	M_n Max	M_p Max	M_p final	M_p initial	M_n initial
Line 2	2.4 x10^{-5}	-	12857	6745	7456	6893
Line 3	7.4 x10^{-4}	14792	16212	6975	7456	6893
Line 4	2.4 x10^{-3}	17287	21906	6989	7456	6893
Line 5	7.4 x10^{-3}	20048	27382	6989	7456	6893
